# The hurdle with remaining risk for hepatocellular carcinoma in cirrhotic patients after a hepatitis C cure

**DOI:** 10.1186/s41124-016-0019-3

**Published:** 2016-09-23

**Authors:** Soo Aleman

**Affiliations:** Department of Gastroenterology and Hepatology, and Infectious Diseases, Karolinska University Hospital/Karolinska Institutet, Huddinge, Sweden

## Abstract

After introduction of new direct acting antivirals (DAAs) against hepatitis C, the cure rate has increased substantially especially in patients with liver cirrhosis. Decreased but remaining risk for hepatocellular carcinoma (HCC) has been shown in patients with liver cirrhosis after cure, in previous studies with interferon (IFN)-based treatments. This risk for HCCs is expected to become the next hurdle in the management of hepatitis C patients, as the number of treated and cured patients with liver cirrhosis is increasing dramatically. At the recent International Liver Congress 2016, Barcelona, Spain, a potentially alarming report was presented by Buonfiglioli F et al., among otherwise positive reports, for patients with prior HCC being treated with DAAs. This preliminary report showed a high early recurrence rate of 29 % for HCC after initiation of DAA treatment in patients with treated HCC, at follow-ups 12–24 weeks post-treatment. Another study was published just prior to this report by Reig M et al. showing similarly high recurrence rate for HCC. In this study, patients who have been treated for HCC with ablation, resection or transarterial chemoembolization, and no sign of remaining HCC at treatment start, were analysed for the risk of HCC recurrence after DAA treatment initiation. After a median follow-up time of 5.7 months, recurrence rate of HCC was seen in 28 %. The disadvantage of these studies was the lack of any control group, but these figures were unexpectedly high compared to figures in previous studies. These findings need to be further explored and eventually confirmed in other studies before making any firm conclusions and change of the routine practice. Until we have more data, the eventual risks for early HCC recurrence and other risks must be weighed against other benefits of these DAAs, halting liver disease progression, on an individual basis.

## The progress of hepatitis C treatment

A true revolution within treatment of hepatitis C has taken place in the last few years with the launch of new direct acting antivirals (DAAs). The cure rate has increased substantially to >95 % compared to previous treatments based on interferon (IFN), which had lower cure rates of 50 %. Frequent side-effects and even serious or fatal, could occur in patients with advanced liver disease with IFN based treatments, i.e.in the group with most need of a cure. Nowadays, we can treat those with decompensated liver cirrhosis, which were previously left out of treatment, with high cure rates and with a generally good safety profile [1].

One of the main goals with hepatitis C treatment in patients with liver cirrhosis to prevent further disease progression and decrease the risk for hepatocellular carcinoma (HCC), and thereby prevent liver-related deaths. Previous studies with IFN-based treatments in patients with compensated liver cirrhosis have shown that progression to decompensation could be stopped in most cases with increased survival [2, 3]. The biggest problem was though that the risk for HCC could not be abolished totally, but remained at approximately 1 % per year [2, 3]. Given the usually aggressive nature and late symptoms of HCC, the surveillance for HCC with ultrasound every 6 months, which was standard practice before cure, needed to continue after the end of treatment [4–6]. This resource consuming practice was maybe not such a big hurdle in the IFN era, since the number of cirrhotic patients who actually achieved cure was not very high, due to poor tolerability or contraindications to treatment and the limited efficacy. Also, the significant mortality in cirrhotics without cure from chronic hepatitis C, meant fewer patients needing surveillance for HCC.

This excellent efficacy together with the other benefits paved the way for the high costs of DAAs, which in turn led to restrictions on the availability for treatments of hepatitis C. Even in most high-income countries, restrictions on who to treat remain, but the patient group with liver cirrhosis has certainly been one of the most prioritized groups for treatment, in accordance with international guidelines [4, 5]. The number of cirrhotics achieving cure after treatment has taken a sharp turn upwards since the introduction of DAAs. This, together with strongly decreased risks to die of liver failure, will therefore lead to substantially increased number of cirrhotic patients with cure, who need to undergo surveillance for the remaining risks of HCC. This issue is therefore expected to be the next hurdle in the management of hepatitis C patients. The continued surveillance programme will be health care resource consuming, and the anxiety or fear for diagnosis of HCC among patients can impact their quality of life. If most patients could be treated and cured before reaching the stage of cirrhosis in the future, this would no longer be an issue.

## Can DAA treatments cause harm in certain hepatitis C patients?

The hepatitis C news from international liver conferences around the world in recent years has been quite euphoric for health professionals and patients. Extensive presentation of one treatment breakthrough after another, with efficacy results sky-rocketing even in the previously most difficult-to-treat patient groups has taken place. The ¨almost too good to be true¨ study results of randomized controlled trials regarding efficacy, adverse events and safety profile of DAAs have now been validated in large real-life cohorts [7, 8]. At the recent EASL International Liver Congress 2016, a potentially alarming report was, however, presented by Italian colleagues, Buonfiglioli F et al. [9], among otherwise positive reports, for patients with prior HCC being treated with DAAs. Prior to this conference, some concerns about the safety o f treating the patients with the most advanced liver disease, i.e. Child Pugh class C patients or those with high MELD scores >18 have been raised, and also whether they would really benefit from DAA treatments before liver transplantation. Few patient cases with reactivation of hepatitis B have been reported in patients treated with DAAs [10]. I have in the recent year heard from Swedish and international colleagues of anecdotal patient cases, in which explosively growing HCCs were detected early after treatment initiation or achievement of cure, but wondered if these were not just observations due to consequence of treating now more liver sick patients.

In the study by Buonfiglioli F et al., 344 hepatitis C infected patients with Child Pugh class A or B liver cirrhosis underwent ultrasound with contrast at treatment start, and at follow-ups of 12 and 24 weeks post-treatment with DAAs. At these short follow-ups, 3.2 % were diagnosed with HCC among patients with no history of previous HCC, while 29 % of patients with history of prior HCC were diagnosed with recurrent HCC. The disadvantage of the study was the lack of any control group, but these figures, especially in those with a history of previous HCC, appeared to be unexpectedly very high. Whether the risk of 3.2 % for HCC in those with cirrhosis without prior HCC was higher than or as expected, is difficult to speculate. This is due to the diverse incidence of HCC in patent groups depending on the severity of cirrhosis, with expected higher figure in those with Child Pugh class B/C cirrhosis. Two days prior to this presentation, a study by Reig M et al. from Spain was published online, raising the same concern of whether we can actually harm certain patient groups by treating them with DAAs [11]. Fifty-eight patients, who were previously treated for HCC with ablation, resection or transarterial chemoembolization and had complete response, were evaluated for HCC recurrence after DAA treatment initiation. The median follow-up time was 5.7 months. 28 % were diagnosed for recurrent HCC, with median time for these diagnoses of 3.5 months after DAA treatment start. This recurrence rate was much higher than expected, comparing to other published studies [12]. A possible mechanism of immune cancer surveillance being disrupted by the fast reduction of inflammation by DAAs, leading to progression of dormant tumor clones to HCCs has been proposed [11].

These new reports contained alarm signals not observed in the IFN era. These findings need to be further explored and eventually confirmed in other studies before making any firm conclusions and change of the routine practice. The European Medicines Agency (EMA) has previously initiated a review to assess the extent of hepatitis B reactivation in patients treated with DAAs for hepatitis C, and will now also extend it to include the risks for early HCC recurrence [13].

In summary, the introduction of DAAs against hepatitis C has led to substantially increased number of cured cirrhotics with continued need of surveillance for HCC, and this issue is expected to become the next hurdle in the management of these patients. The potentially increased risks for HCC recurrence in those with prior HCC after treatment with DAAs, as shown in few alarming reports, must be weighed against other benefits of the treatment with halting the progression of liver disease, on an individual basis until we have more data.

## Abbreviations

DAAs: New direct acting antivirals
EMA: The European Medicines Agency
HCC: Hepatocellular carcinoma
IFN: Interferon
